# A Dual Role of Strigolactones in Phosphate Acquisition and Utilization in Plants

**DOI:** 10.3390/ijms14047681

**Published:** 2013-04-09

**Authors:** Olaf Czarnecki, Jun Yang, David J. Weston, Gerald A. Tuskan, Jin-Gui Chen

**Affiliations:** Biosciences Division, Oak Ridge National Laboratory, Oak Ridge, TN 37831, USA; E-Mails: czarneckio@ornl.gov (O.C.); yangj3@ornl.gov (J.Y.); westondj@ornl.gov (D.J.W.); tuskanga@ornl.gov (G.A.T.)

**Keywords:** arbuscular mycorrhizal fungi (AMF), phosphate (Pi), phosphorus (P), root development, shoot branching, strigolactones (SLs)

## Abstract

Phosphorus, acquired in the form of phosphate (Pi), is one of the primary macronutrients for plants but is least available in the soil. Pi deficiency is a major factor limiting plant growth, development and reproduction. Plants have developed a complex signaling network to respond to Pi deficiency. The recent discovery of strigolactones, a new class of plant hormones, has led to an emerging signaling module illustrating the integrated control of Pi acquisition, plant-microbe symbiotic interactions and plant architecture. This review article focuses on the recent findings of plant responses and roles of strigolactones to Pi deficiency.

## 1. Introduction

Unlike animals, plants are sessile and are constantly subjected to environmental challenges. As a consequence, plants have evolved complicated yet precise mechanisms to respond and adapt to their environment. One recent breakthrough in the field of plant biology was the discovery of strigolactones (SLs) as a new class of plant hormones controlling shoot branching [1,2]. It appears that synthesis and exudation of SLs is regulated by nutrient availability in soil [3–5] and SLs exuded by roots serve as host recognition signals for symbiotic fungi [6]. Therefore, SLs are viewed as integrative signaling molecules coupling nutrient availability and microbial symbiosis to plant architecture and productivity. In this article, we will present a brief overview of the current knowledge on phosphate (Pi) deficiency in plants followed by a detailed discussion of how SLs help plants manage Pi deficiency.

## 2. Phosphorus Availability Is a Major Factor Limiting Plant Growth, Development and Reproduction

Phosphorus (P) is an essential plant nutrients and is incorporated in numerous metabolites including sugar phosphates, nucleotides and nucleic acids, phospholipids, phosphoproteins and energy-rich compounds like adenosine triphosphate (ATP). In contrast to other essential nutrients such as carbon (C) and nitrogen (N) that can be fixed from the atmosphere, the only natural source of P is weathering of minerals in soils or atmospheric inputs [7]. P concentrations in crop plants range from 0.15% to 0.5% dry weight [8], but since typical Pi concentrations are below 10 μM in soils [9] or 1–3 μM (dissolved Pi) in oceans [10], its availability in both terrestrial and aquatic ecosystems is limited. The effects of soil fertilization are reduced because 80% of the applied P adsorbs to clays and Al/Fe oxides, precipitates and forms Ca, Fe and Al phosphates, or is converted to organic phosphates (e.g., phytic acid) that are not immediately available for plants [11–14]. Moreover, sources of P ores are non-renewable and are being rapidly depleted by usage [15,16]. It is generally considered that N limitation has a greater impact on biomass formation, directly followed by P. However, with increasing soil age, particularly in tropical or subtropical regions, P limitation tends to become the major limiting factor of plant productivity [17,18].

Inorganic P (Pi) is the main P-form that plant roots can absorb and after uptake, it either remains as Pi or is assimilated by forming an ester with a hydroxyl group of a carbon chain (e.g., sugar phosphates) or attaches to another Pi by forming an energy-rich pyrophosphate bond (e.g., ATP) [14]. Typical symptoms of Pi starvation in plants include stunted growth, dark green leaf colors caused by accumulation of anthocyanins and leaf necrosis [19]. Lack of Pi usually correlates with decreased levels of ATP, photosynthetic activity and stomatal conductance [20–24] that all result in reduced biomass production. Under Pi deficient conditions, however, plants have evolved several strategies to enhance Pi acquisition and reduce the need for Pi to act on multiple levels, including transcriptional activation, biochemical adaptations, physiological responses and morphological changes.

## 3. Morphological, Physiological and Biochemical Responses of Plants to Pi Deficiency

**Figure 1:**
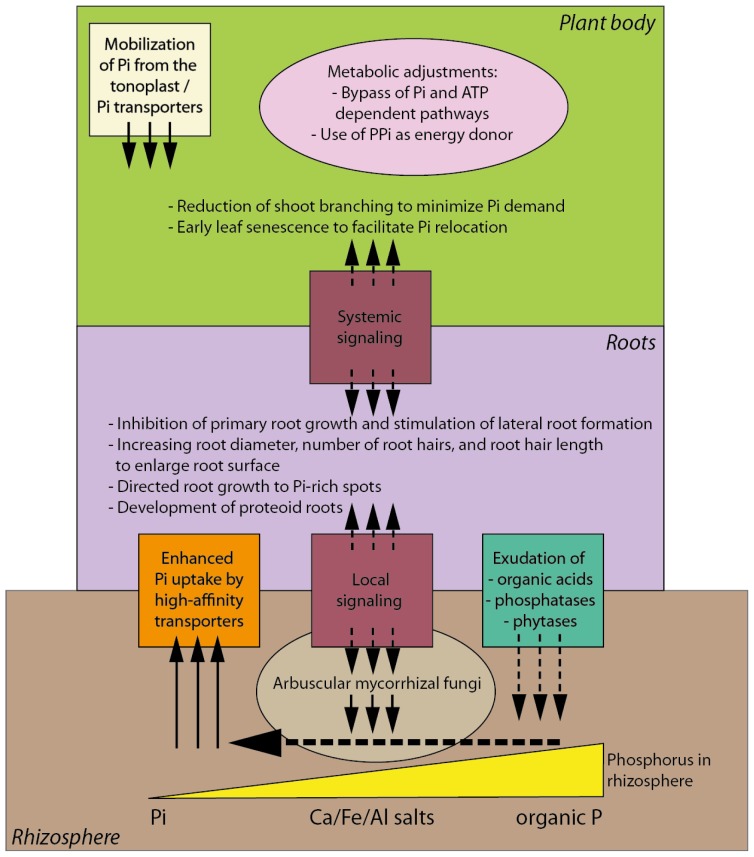
Conceptual summary of morphological, physiological and biochemical responses to phosphate (Pi) deficiency in plants. Besides the listed morphological changes in root architecture, plant roots facilitate Pi uptake by expression of high affinity Pi transporters and by exudation of organic acids, phosphatases and phytases to mobilize additional Pi resources. The tonoplast plays an important role to maintain Pi homeostasis during Pi deficient periods. Long-term Pi deficiency is compensated by metabolic adjustments in order to lower Pi and ATP demands. A complex network of local and systemic signaling pathways is involved in plant response to Pi deficiency (details are given in the text) and strigolactones may act both at the local signaling and the systemic signaling levels.

Modifications of root growth and architecture are the most obvious and best-documented responses of plants to Pi starvation [25–27]. Under Pi deficient conditions, root diameter decreases and the number of root hairs as well as their length increases which help enhance Pi uptake by increasing soil surface area contact [25,28–34] (Figure 1). The importance of strategic root growth adjustments to compensate for low Pi availability is underlined by directed root growth towards Pi-rich pockets within soils [35,36]. As will be discussed in details below, mycorrhizal-mediated mineral acquisition plays an important role in supplying nutrition for plants [37,38].

Another root-related response to Pi starvation is the formation of proteoid roots (or cluster roots) in certain plant species, such as lupine and protaceae [39]. These clusters of rootlets are induced by rhizosphere bacteria and are specialized in the synthesis and excretion of organic acids (e.g., citrate) that help mobilize Pi from Ca, Fe and Al salts [16,40–43]. Exudation of organic acids from roots is not limited to proteoid roots but is a more general strategy to solubilize Pi by chelating Ca, Fe and Al cations [25,44]. In addition, secretion of phosphatases or phytases by roots increases mobilization of Pi bound to organic components (e.g., phytic acid) that are considered to form the greatest phosphorus pool in soils [45–51].

The uptake of soluble or mobilized soil Pi needs to occur against a relatively high concentration gradient because the cytoplasmic Pi concentrations usually exceed Pi concentration in soil by an order of magnitude. Pi is likely absorbed in the most abundant forms, H_2_PO_4_^−^ or HPO_4_^2−^, by high affinity Pi/H^+^ symporters that belong to the *PHOSPHATE TRANSPORTER1* (*PHT1*) gene family; a process requires an H^+^-ATPase driven proton gradient across the plasma membrane [12,52–54]. PHT1, PHT2 and PHT3 transporters also play important roles in intracellular Pi translocation or transport between plant organs [55]. Up to 95% of the plant’s Pi is stored in the vacuole [24,25], which serves as a buffer to maintain stable cytoplasmic Pi concentrations under varying levels of available Pi [56]. However, the tonoplast is unable to sufficiently fulfill the cellular Pi demand under prolonged Pi starvation [57]. Pi starvation induces the expression of high-affinity Pi transporters increasing the potential for enhanced Pi uptake from soils [12,13,55].

Besides the physiological and morphological adaptations, Pi starvation also causes striking metabolomic changes in plants (Figure 1). In general, metabolic redundancies allow activation of enzymes that do not require ATP or Pi as substrates, such as enzymes of the glycolytic and an alternate respiratory pathway, or replacement of phospholipids with nonphosphorus galactolipids in extraplastidic membranes [22,51,58–60]. Pi deficiency-caused reduction in ATP and ADP levels inhibits glycolytic enzymes and Pi- and adenylate-independent enzymes that bypass classical glycolysis are up-regulated [61]. Some of these enzymes use pyrophosphate (PPi) as an energy donor, which also helps recycle Pi and restore ATP. PPi is formed as a byproduct of a wide range of biosynthetic reactions (e.g., polymerization reactions) and is present at high concentrations of up to 300 μM in the cytosol [62]. Pi starvation induces PPi-dependent phosphofructokinase (replaces ATP-dependent phosphofructokinase), phosphoenolpyruvate carboxylase and pyruvate Pi dikinases (replace ADP-dependent pyruvate kinase) (reviewed in [51,55]).

## 4. Complex Signaling Network Underlying Plant Response to Pi Deficiency

Although a complete picture of how plants sense external (and internal) Pi concentrations and transduce this information into the abovementioned morphological, physiological and biochemical responses is still lacking, the past decade has brought significant progress towards the understanding of the complex signaling pathways underlying plant responses to Pi deficiency. Here we only briefly describe the major components of the signaling network responsible for sensing and responding to Pi starvation. For a comprehensive overview of signaling networks related to Pi availability, readers are referred to recent review articles [63–67].

In general, the Pi-deficiency signaling network can be subdivided into two parts, (1) local and (2) systemic or long distance signaling. Sensing of external Pi concentrations likely takes part at the root tip and induces changes in root architecture. There is evidence to support that Pi itself acts as signaling molecule [68,69]. Analysis of *phosphate deficiency response* 2 (*pdr2*), an *Arabidopsis* mutant with exaggerated Pi-deficient root phenotype resulting from an impaired P5-type ATPase, as well as mutants with altered sensitivity to Pi starvation [*low phosphorus insensitive* (*lpi*), *low phosphate root 1* (*lpr1*), *lpr2* and *Pi starvation-induced* (*psi*)] revealed that inhibition of primary root growth accompanied by lateral root formation in response to low external Pi is likely regulated by cells within the root tip [70–74]. There is a genetic interaction between PDR2 and the multicopper-oxidases LPR1/PSI and LPR2 where PDR2 acts upstream and negatively regulates the responses of LPR1/PSI and LPR2. Moreover, PDR2, located in the endoplasmic reticulum, is involved in the expression regulation of SCARECROW, a transcription factor regulating root patterning and stem cell maintenance. Although a detailed model depicting the local signaling pathway from sensing external Pi concentrations to transcriptional responses in root tips is still lacking, it has been shown that this process is dependent on the auxin receptor TRANSPORT INHIBITOR RESPONSE1 (TIR1) [75,76] and MORE AXILLARY GROWTH 2 (MAX2), a key signaling component in the strigolactone pathway (further discussed below).

Several systemic signaling pathways have been proposed to be involved in Pi-deficiency signaling and it is likely they act in an interconnected manner [63,66]. There is evidence to support a role of plant hormones in Pi signaling. For example, auxin and ethylene treatments and Pi deficiency cause similar phenotypic changes in plant roots [32,77]; endogenous levels of cytokinin and cytokinin receptors decrease under Pi-starvation and reduction in cytokinin concentrations is a prerequisite for a proper Pi starvation response [78], and gibberellins can repress root morphological changes under Pi starvation via a DELLA transcription factor-dependent signaling mechanism [79]. Because sugar signaling is closely related to multiple plant hormones [80], it is not surprising that crosstalk also exists between Pi status and sugar signaling [81].

One of the better-understood Pi-dependent systemic signaling pathways involves the micro-RNA miR399. PHOSPHATE 2 (PHO2) is an ubiquitin-conjugating enzyme likely involved in targeting the abovementioned PHT Pi transporters for proteolytic degradation. Micro-RNA miR399 not only directs the cleavage and degradation of *PHO2* mRNA, it also acts as a systemic signal moving from shoots to roots via the phloem. Under Pi-deficient conditions, expression of miR399 is strongly induced resulting in down-regulation of *PHO2* mRNA, decreased proteolytic degradation of PHT proteins and enhanced Pi uptake [82–86]. Recently, it has been demonstrated that SLs act as long-distance signals to transport from roots to shoots (e.g., opposite direction to the movement of miR399) during Pi deficiency to inhibit shoot branching to help plants adapt to Pi deficient conditions by reducing Pi utilization but also to enhance Pi acquisition through stimulation of symbiotic interactions with arbuscular mycorrhizal fungi (AMF). In the second part of this review, we specifically focus on discussing the recent progress indicating SL as a key signaling molecule in plants under Pi deficiency stress.

## 5. Strigolactones: Physiological Roles, Biosynthesis and Signaling

**Figure 2:**
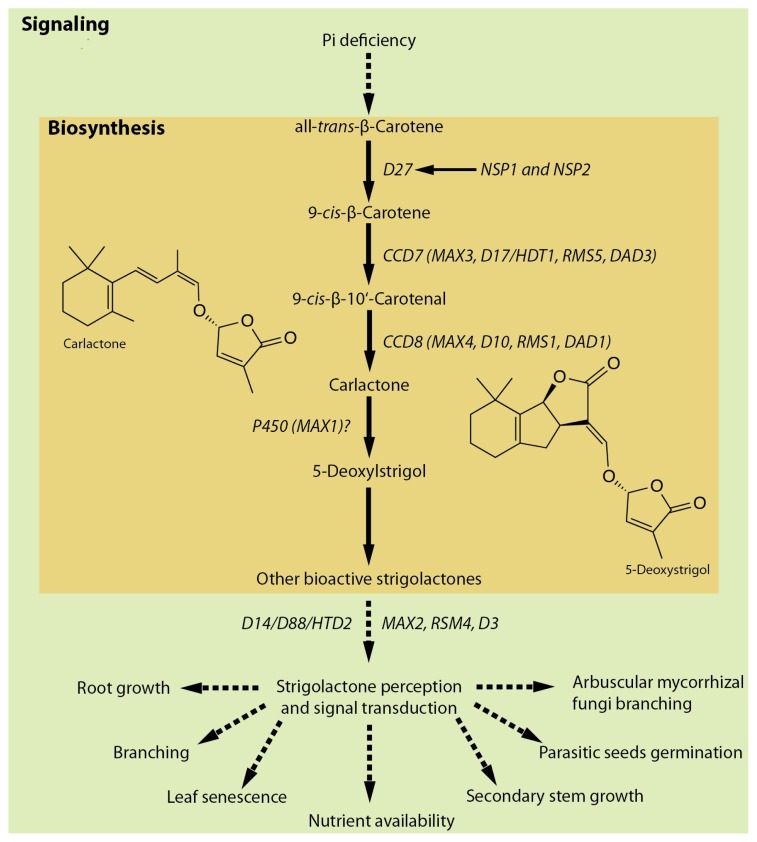
Summary of strigolactone biosynthetic and signaling pathways. The biosynthesis involves two carotenoid cleavage dioxygenases, CCD7 (MAX3, RMS5, D17/HTD1, DAD3) and CCD8 (MAX4, RMS1, D10, DAD1), one cytochrome P450 monooxygenase (MAX1) and one novel iron-containing protein (D27) [88,93,102–107]. In *Medicago*, SL biosynthesis also requires GRAS-type transcription factors NODULATION SIGNALING PATHWAY1 (NSP1) and NSP2 [108]. SL signaling involves MAX2/RMS4/D3 [109], an F-box leucine-rich protein and DWARF14 (D14)/DAD2/D88/HTD2 [110–112], a member of α/β-fold hydrolase superfamily. Potential downstream component in the SL pathway, such as members of the TCP family of transcription factors, are not shown in the chart.

SLs are terpenoid lactones derived from carotenoids [87,88], which were originally isolated from plant root exudates and recognized as germination stimulants for root parasitic plants such as *Striga*, *Orobanche* and *Phelipanche*[89]. Subsequently, SLs were also identified as stimulants of hyphal branching and root colonization of symbiotic AMF [6,90]. More recently, it has been demonstrated that SLs act as long-distance signaling molecules that can be transported from roots to shoots to exert its specific functional control on shoot branching [1,2,91]. The transport of SLs from roots to shoots is partly mediated by ATP-binding cassette (ABC) transporters [91,92]. In addition to their founding role acting as host recognition signals for parasitic weeds and AMF and their newly-discovered role acting as a key suppressor of shoot branching, recent studies demonstrate that SLs also regulate many other processes in plant growth and development including primary root growth, lateral root formation, adventitious root formation, root hair development, seed germination, photomorphogenesis, stress response, nodulation and protonema branching in moss (Figure 2). These other aspects of SL physiological roles and signaling have been thoroughly covered by a number of recent review articles [93–101] and readers are strongly encouraged to refer to these articles and references therein for details. Here we only briefly describe the key components involved in SL biosynthesis and signaling.

Genetic analysis of branching mutants has been particularly important in uncovering genes involved in SL biosynthesis and signaling pathways. These include *more axillary growth* (*max*) mutants of *Arabidopsis*, *ramosus* (*rms*) mutants of *Pisum sativum*, *dwarf* (*d*) or *high tillering dwarf* (*htd*) mutants of *Oryza sativa* and *decreased apical dominance* (*dad*) mutants of *Petunia*. Analysis of these mutants has revealed that SL biosynthesis involves two carotenoid cleavage dioxygenases, CCD7 (MAX3, RMS5, D17/HTD1, DAD3) and CCD8 (MAX4, RMS1, D10, DAD1), one cytochrome P450 monooxygenase (MAX1) and one novel iron-containing protein (D27) [88,93,102–107]. In *Medicago*, it was shown that SL biosynthesis also requires the GRAS-type transcription factors NODULATION SIGNALING PATHWAY1 (NSP1) and NSP2 that are essential for rhizobium Nod factor-induced nodulation [108].

Grafting experiments have demonstrated that MAX2/RMS4/D3, an F-box leucine-rich protein [109], is an important signaling component of the SL pathway. In other plant hormone signaling pathways (e.g., auxin, gibberellin and jasmonic acid), ubiquitin-mediated protein degradation of negative regulators is a key regulatory step, which involves F-box proteins [113]. It is likely that a similar process is operating in the SL signaling pathway via MAX2. Rice *dwarf14* (*d14*) is an SL-insensitive mutant that displays accelerated outgrowth of tiller phenotype [110]. Similarly, a loss-of-function mutation in the *Arabidopsis D14* ortholog, *AtD14*, also conferred increased shoot branching and SL-insensitivity [111]. *D14* encodes a protein of the α/β-fold hydrolase superfamily. Because some members of this family, such as GID1 [114], have been found to act as a receptor for plant hormones, these findings raise the possibility that D14 may be a component of an SL receptor complex. Computational-based structure analysis using homology modeling and molecular dynamic simulation and crystal structure analysis support this view [115,116]. More recently, it was found that PhDAD2, a *Petunia* ortholog of D14, interacts with PhMAX2A in a GR24 (a synthetic SL analog) concentration-dependent manner [112]. Furthermore, DAD2 binds and hydrolyzes GR24 and a mutation in the catalytic triad of DAD2 abolished both its hydrolase activity and its ability to interact with PhMAX2A. It has also been demonstrated that D14 can directly bind GR24 [117]. These studies provide strong evidence to support the view that DAD2/D14 is a part of SL perception complex and that DAD2/D14 is a SL receptor itself. One potential downstream component in the SL signaling pathway is FINE CULM1 (FC1), which is a member of the TCP transcription factor family [118,119]. Consistent with this view, it recently was found that the *Pisum sativum* TCP transcription factor PsBRC1, a homolog of the maize TEOSINTE BRANCHED1 [120] and the *Arabidopsis* BRANCHED1 (AtBRC1) [121], acts downstream of MAX2 to control shoot branching [122].

Because the topics on the physiological roles of SLs have been thoroughly covered by a number of recent review articles [93–101], we will not reiterate them here. Instead, we briefly summarize the roles of SLs in three processes that will be further discussed below relevant to Pi deficiency: (1) Shoot branching: SLs function as negative regulators of shoot branching, which is supported by large amount of genetic studies using SL-deficient and SL-responsive mutants as well as the studies using GR24 in a number of plant species (summarized extensively in abovementioned review articles); (2) Hyphal branching in AMF: SLs function as positive regulators in this process, which has been demonstrated both chemically where hyphal branching can be directly stimulated by both root-exuded SLs and GR24 [6] and genetically where plants deficient in SL showed reduced mycorrhizal colonization of the root and GR24 application enhanced mycorrhizal development in both wild-type plant and SL-deficient mutants [1,92,123]. However, under high Pi conditions, SLs alone are not sufficient to enhance mycorrhizal colonization in some plant species [123–125], suggesting the existence of additional signals controlling the differentiation of hyphopodia. (3) Root development: Compared with shoot branching in which SLs play a major role, the roles of SLs in the regulation of root development are relatively moderate. Nonetheless, accumulating evidence supports that SLs modify every aspect of root architecture including primary root growth, lateral root formation, adventitious root formation and root hair development. For example, the length of primary roots of *Arabidopsis* SL-deficient and -insensitive mutants are shorter due to a reduction in meristem cell number, which could be rescued by application of GR24 in all genotypes except in the SL-insensitive mutant [126]. Under Pi-sufficient conditions, SL-deficient and -insensitive mutants displayed increased lateral root density than wild-type [127]. GR24 application suppressed lateral root primordial development and lateral root forming potential, leading to a reduction in lateral root density and number of lateral roots in SL-deficient mutants but not in SL-insensitive mutants [126,127]. Under low Pi conditions, SL-deficient and SL-insensitive mutants display reduced root hair density compared with wild-type and GR24 treatment can recover such defects in SL-deficient mutant, but not in SL-insensitive mutant [75]. GR24 treatment also led to an increase in root-hair length in wild-type and SL-deficient mutants, but not in SL-insensitive mutants [127]. Furthermore, the treatment of SL biosynthesis inhibitor AbamineSG reduced the root hair density of wild-type plants to levels that were similar to those in non-treated SL-deficient mutants [75]. Finally, both SL-deficient and SL-signaling mutants showed enhanced adventitious roots formation and GR24 treatment suppressed adventitious roots formation in wild-type plants [128]. In summary, SLs inhibit primary root growth, lateral root formation and adventitious root formation and promote root hair formation. Because the effect of SLs on modification of root system is relatively mild, it is likely that SLs play a modulatory role in this process by modulating signaling of other plant hormones such as auxin and ethylene, which are known to be major regulators of root system.

## 6. Pi Deficiency Stimulates SL Biosynthesis and Exudation

The biosynthesis of SLs is regulated by both abiotic and biotic factors. Levels of SLs in roots are significantly increased in a number of plant species when plants are grown under Pi deficient conditions [2–5,129,130]. While Pi starvation-dependent stimulation of SL production appeared to be specific in red clover [4], in sorghum, 5-deoxystrigol content was increased by both Pi and N deficiency [3]. Consistent with the view that Pi deficiency stimulates SL production, the expression levels of genes encoding enzymes involved in SL biosynthesis depend on Pi status. Microarray analyses of transcriptional changes associated with elevated Pi supply in *Petunia* revealed that genes encoding enzymes involved in carotenoid and apocarotenoid biosynthesis are generally suppressed and the expressoion of *DAD1* encoding SL biosynthetic enzyme CCD8 was suppressed [123].

Because Pi deficiency stimulates SL biosynthesis, one would expect that similar phenotypic changes are observed in plants grown under Pi deficient conditions and SL-treated plants. It was found that elevated SL levels in plants grown under Pi deficient conditions are normally accompanied by a suppression of bud outgrowth and shoot branching [2–5,91,129], one of the best characterized phenotypic traits regulated by SLs. Furthermore, the suppression of *Oryza sativa* tiller bud outgrowth [5] and *Arabidopsis* shoot branching [91] under Pi deficient conditions does not occur in SL-deficient and -insensitive mutants. These findings support the view that Pi deficiency stimulates SL biosynthesis which in turn modifies plant growth.

In addition to stimulating *de novo* SL biosynthesis, Pi deficiency can also stimulate the exudation of SLs from roots. After biosynthesis, some SLs are readily available to be exuded into the rhizosphere. It has been shown that SL exudation is negatively correlated with Pi supply in *Trifolium pratense*[4] and *Solanum lycopersicum*[129,131]. When Pi starved plants were transferred to medium containing sufficient Pi, SL exudation dropped within 24 h [4]. Umehara *et al.*[5] demonstrated a negative correlation between SL concentration in root exudates and the number of outgrowing tillers in *Oryza sativa*. In this case, the SL levels in root exudates gradually decreased and the number of outgrowing tillers increased in response to increasing concentrations of Pi in the media [5].

Initially, it appeared that in nodulating leguminous plants, SL exudation is promoted only under Pi deficiency whereas in other mycotrophic plants it occurs under both Pi and N deficient conditions. For example, in *Trifolium pratense*, Pi deficiency, but not N deficiency, promotes SL exudation [4] whereas in *Sorghum* plants, both Pi and N deficiency could enhance 5-deoxystrigol and sorgomol exudation [3]. Similarly, in *Oryza sativa*, both Pi [5] and N deficiency [5,132] promoted SL exudation. However, an examination and comparison of SL exudation between a number of legumes and non-legumes indicated that plant response to SL exudation to nutrient availability does not directly relate to the plant’s ability to form symbiotic relationships with root nodule bacteria [133]. It was proposed that N deficiency alters SL production and exudation, mainly by altering Pi levels in shoots [133]. When shoot Pi levels were low, SL exudation was promoted; when shoot Pi levels were high, SL exudation was suppressed in plants grown under N deficient conditions, implying that N deficiency may regulate P metabolism and/or P transport from roots to shoots.

Yoneyama *et al.*[134] analyzed the root exudates in low Pi condition from 13 Asteraceae species and found that, except for *Helianthus annuus*, all plants examined exuded known SLs. Orobanchyl acetate and orobanchol were the two major SLs exuded by these Asteraceae species but 5-deoxystrigol and 7-hydroxyorobanchyl acetate were also detected in root exudates from several species. These findings implied that a general SL biosynthesis pathway is conserved in these species but it may involve species-specific modifications.

The association of AMF with plant roots is one of the most important symbiotic relationships between plants and microorganism. Approximately 80% land plants form symbiotic association with AMF [135]. The symbiotic fungi obtain water and nutrients, primarily Pi and N, from the soil and translocate them to the host plants helping their growth and development [6,136]. In return, AMF obtain carbohydrates from the host plants to promote their growth. AMF spores treated with root exudates from plants grown under Pi starvation were found to have more hyphal branching activity than those treated with exudates from Pi sufficient plants [137]. Moreover, increased soil Pi levels resulted in a decreased AMF colonization of the roots [135]. SLs exuded from roots are perceived by AMF and induce hyphal branching, a step necessary to establish mycorrhizal symbiosis [6]. This view is supported by recent genetic studies in which SL-deficient plants were found to display reduced mycorrhizal colonization of the roots and GR24 treatment enhanced mycorrhizal development in both SL-deficient mutants and wild-type plants [1,92,123]. Since AMF helps plants acquire Pi from soil, the exudation of higher levels of SLs under Pi starvation conditions is a beneficial strategy for plant adaptation and survival [4]. Little is known about how SLs are perceived by AMF resulting in stimulation of spore germination and hyphal branching. This process involves the activation of mitochondria [90]. It was shown that SLs stimulate cell proliferation of the AM fungus Gigaspora, increase the density and movement of mitochondria [90] and rapidly activate oxidative metabolism [138]. Within the host plants, it was found that the F-box protein, DWARF3 (D3) which is an ortholog of MAX2, but not D14 or D14-like, is required for establishing AM symbiosis [139]. Because D14 acts as a receptor for SLs [112,115–117], this finding demands further investigation on SL signaling pathway in AM symbiosis, both in the host and in the AMF.

The exudation and transport of SLs require PhPDR1, an ATP-binding cassette (ABC) transporter which has been recently identified as a cellular SL exporter in *Petunia hybrida*[92]. PhPDR1 is predominantly localized in the plasma membrane of individual sub-epidermal cells of lateral roots [92]. *pdr1* mutants are defective in SL exudation from roots and such plants display reduced AMF symbiosis. The expression of *PhPDR1* in roots was induced by Pi starvation, AMF colonization and GR24 treatment. Overexpression of *PhPDR1* in *Arabidopsis* resulted in an increased tolerance to high concentrations of GR24, consistent with increased export of SLs from the roots. Interestingly, the closest homolog of PhPDR1 in *Arabidopsis* is ABCG40 that transports abscisic acid [140], a plant hormone that is also derived from carotenoids.

It should be noted that the stimulation of SL biosynthesis and exudation by Pi deficiency is also conserved in plants that are not hosts for AMF, e.g., *Arabidopsis*[91]. The physiological function of root-exuded-SLs by non-AMF-host plants remains unclear. It has been speculated that this may be a relic of an ancestral trait of most land plants [91]. It is also possible that root-exuded-SLs may act as a signal for other unidentified microorganisms. It should also be noted that in some plant species, such as *Lupinus albus*[130], Pi deficiency does not lead to stimulation of SL exudation. On the other hand, application of GR24 failed to increase mycorrhizal colonization in *Pisum sativum* and *Petunia* under high Pi conditions [123,124]. Further, *Pisum sativum* SL-insensitive mutant *rms4* was still able to respond to Pi levels during mycorrhizal colonization [125]. These findings suggested that SLs are not the only limiting factor for mycorrhizal colonization which may require additional stimulators. There may be a species-specific mechanism underlying stimulation of SL exudation by Pi deficiency and SLs may have other roles yet to be described.

## 7. Model: A Dual Role of Strigolactones in Pi Acquisition and Utilization under Pi Deficiency Conditions

**Figure 3:**
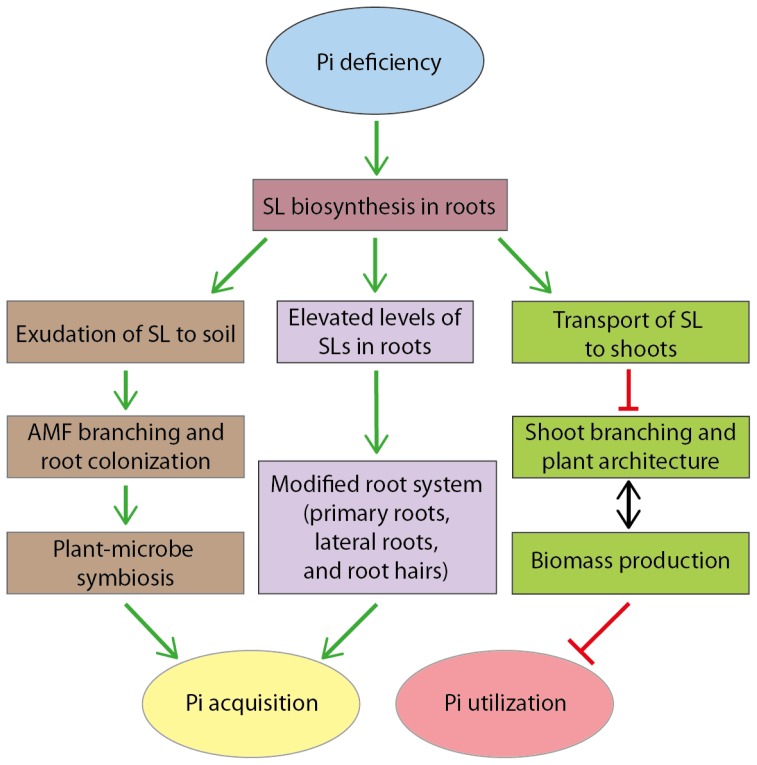
A model illustrating a dual role of strigolactones (SLs) in the modulation of Pi acquisition and utilization under Pi deficient conditions. Under Pi deficiency, plants need to minimize the production of new shoot branches and direct limited Pi resources to already existing shoots while maximizing Pi acquisition from soil. Pi deficiency stimulates SL biosynthesis in roots and exudation to soil. Elevated SLs (acting as endogenous hormones) act locally by modifying root system to increase root coverage that provides more surface area to explore more soil volumes and allow higher Pi uptakes. SLs are also transported through the xylem to suppress shoot branching [91], a means to reduce Pi utilization. SL exudation to soil serves as rhizosphere signal for symbiotic interaction between some host plants and arbuscular mycorrhizal fungi (AMF), a means to increase Pi acquisition. Note: approximately 80% land plants form symbiotic association with AMF.

Under the current working conceptual model, Pi deficiency stimulates SL biosynthesis and exudation in roots. Elevated levels of SLs can act locally by modifying root architecture for increasing root coverage that provides more surface area to explore more soil volumes and allow higher Pi uptakes. But SLs are also transported through the xylem to suppress shoot branching [91], a means to reduce Pi utilization (Figure 3). SL exudation to soils also serves as a rhizosphere signal for symbiotic interaction between the host plant and AMF, a means to increase Pi acquisition (Figure 3). Therefore, SLs play multiple roles in the adaptation to Pi deficient conditions. When Pi supply is sufficient, lower levels of SLs allow plants to initiate multiple branching events and to sustain the subsequent growth and development of new branches. In contrast, under Pi deficient conditions, higher levels of SLs allow plants to maximize Pi acquisition through modified root system and symbiotic interaction with AMF and to minimize the production of new branches thereby directing the limited Pi resource to already existing shoots and not growth of new branches.

In addition to modifying plant architecture including shoot branching and root development and stimulating symbiotic interaction with AMF to minimize Pi utilization and maximize Pi acquisition, SLs act as a positive regulator of leaf senescence [105,141,142], a phenotypic response also observed in plants grown under Pi deficient conditions [143]. For example, the *Arabidopsis oresara9* (*ore9*) mutant exhibited delayed leaf senescence [141]. Genetic mapping revealed that this phenotype was caused by a single base pair change leading to the early termination of translation of *ORE9* which is identical to *MAX2*[141]. Similarly, mutations in *DWARF3* (*D3*), an *Oryza sativa* ortholog of MAX2/ORE9, led to delay in darkness-induced senescence in leaf [142]. Leaf senescence allows plants to reallocate nutrients from old tissues where nutrients are no longer required to younger tissues where development is still occurring. Therefore, under Pi deficient conditions, SLs act as signaling molecules to mediate multiple levels of morphological, physiological and biochemical responses as part of re-programing its adaptation and survival strategies to help plants manage Pi deficiency.

While all of the above represents an attractive model, many questions remain unanswered: (1) Why is the induction of SL biosynthesis and exudation by Pi deficiency not universally found in all species examined? Do species’ differences reflect alternate nutrient acquisition strategy? (2) What determines the nutrient deficiency specificity (e.g., P deficiency *vs.* N deficiency)? (3) What are other factors required for mycorrhizal colonization? (4) What are the roles of SLs exuded by non-AMF-host plants? (5) What are the exact roles of SLs in root development? How do SLs interact with other plant hormones, such as auxin and ethylene? Does Pi deficiency modify root system solely by regulating SL biosynthesis? (6) How is D3 (an *Oryza sativa* ortholog of MAX2), but not D14 (a receptor for SLs), required for establishing AM symbiosis? Future studies will be needed to address these questions and to provide insights into SLs regulation of Pi deficiency at the mechanistic level. It is apparent that acquiring a comprehensive understanding of SL biosynthesis and its regulation, as well as a complete picture of SL signal transduction network, will require significant efforts across multiple disciplinary fields.

## References

[1] Gomez-Roldan V., Fermas S., Brewer P.B., Puech-Pages V., Dun E.A., Pillot J.P., Letisse F., Matusova R., Danoun S., Portais J.C. (2008). Strigolactone inhibition of shoot branching. Nature.

[2] Umehara M., Hanada A., Yoshida S., Akiyama K., Arite T., Takeda-Kamiya N., Magome H., Kamiya Y., Shirasu K., Yoneyama K. (2008). Inhibition of shoot branching by new terpenoid plant hormones. Nature.

[3] Yoneyama K., Xie X.N., Kusumoto D., Sekimoto H., Sugimoto Y., Takeuchi Y., Yoneyama K. (2007). Nitrogen deficiency as well as phosphorus deficiency in sorghum promotes the production and exudation of 5-deoxystrigol, the host recognition signal for arbuscular mycorrhizal fungi and root parasites. Planta.

[4] Yoneyama K., Yoneyama K., Takeuchi Y., Sekimoto H. (2007). Phosphorus deficiency in red clover promotes exudation of orobanchol, the signal for mycorrhizal symbionts and germination stimulant for root parasites. Planta.

[5] Umehara M., Hanada A., Magome H., Takeda-Kamiya N., Yamaguchi S. (2010). Contribution of strigolactones to the inhibition of tiller bud outgrowth under phosphate deficiency in rice. Plant Cell Physiol.

[6] Akiyama K., Matsuzaki K., Hayashi H. (2005). Plant sesquiterpenes induce hyphal branching in arbuscular mycorrhizal fungi. Nature.

[7] Schlesinger W.H., Bernhard E.S. (2013). Biogeochemistry: An analysis of Global Change.

[8] Epstein E. (1994). The anomaly of silicon in plant biology. Proc. Natl. Acad. Sci. USA.

[9] Bieleski R.L. (1973). Phosphate pools, phosphate pransport, and phosphate availability. Annu. Rev. Plant Physiol.

[10] Paytan A., McLaughlin K. (2007). The oceanic phosphorus cycle. Chem. Rev.

[11] Holford I.C.R. (1997). Soil phosphorus: Its measurement, and its uptake by plants. Aust. J. Soil Res.

[12] Shen J.B., Yuan L.X., Zhang J.L., Li H.G., Bai Z.H., Chen X.P., Zhang W.F., Zhang F.S. (2011). Phosphorus dynamics: from soil to plant. Plant Physiol.

[13] Schachtman D.P., Reid R.J., Ayling S.M. (1998). Phosphorus uptake by plants: From soil to cell. Plant Physiol.

[14] Marschner P (2012). Marschner’s Mineral Nutrition of Higher Plants.

[15] Gilbert N. (2009). Environment: The disappearing nutrient. Nature.

[16] Vance C.P., Uhde-Stone C., Allan D.L. (2003). Phosphorus acquisition and use: critical adaptations by plants for securing a nonrenewable resource. New Phytol.

[17] Ae N., Arihara J., Okada K., Yoshihara T., Johansen C. (1990). Phosphorus uptake by pigeon pea and its role in cropping systems of the Indian subcontinent. Science.

[18] Lambers H., Raven J.A., Shaver G.R., Smith S.E. (2008). Plant nutrient-acquisition strategies change with soil age. Trends Ecol. Evol.

[19] Bennett W.F. (1993). Nutrient Deficiencies & Toxocotoes in Crop Plants.

[20] Dietz K.J., Heilos L. (1990). Carbon metabolism in spinach leaves as affected by leaf age and phosphorus and sulfur nutrition. Plant Physiol.

[21] Miginiacmaslow M., Hoarau A. (1982). Variations in the adenylate levels during phosphate-depletion in isolated soybean cells and wheat leaf fragments. Z. Pflanzenphysiol.

[22] Mikulska M., Bomsel J.L., Rychter A.M. (1998). The influence of phosphate deficiency on photosynthesis, respiration and adenine nucleotide pool in bean leaves. Photosynthetica.

[23] Plaxton W.C. (1996). The organization and regulation of plant glycolysis. Annu. Rev. Plant Physiol.

[24] Natr L. (1992). Mineral nutrients—A ubiquitous stress factor for photosynthesis. Photosynthetica.

[25] Raghothama K.G. (1999). Phosphate acquisition. Annu. Rev. Plant Physiol.

[26] Lynch J. (1995). Root architecture and plant productivity. Plant Physiol.

[27] Williamson L.C., Ribrioux S.P.C.P., Fitter A.H., Leyser H.M.O. (2001). Phosphate availability regulates root system architecture in Arabidopsis. Plant Physiol.

[28] Fohse D., Claassen N., Jungk A. (1991). Phosphorus efficiency of plants. II. Significance of root radius, root hairs and cation-anion balance for phosphorus influx in 7 plant-species. Plant Soil.

[29] Gahoonia T.S., Nielsen N.E. (1998). Direct evidence on participation of root hairs in phosphorus (P-32) uptake from soil. Plant Soil.

[30] Bates T.R., Lynch J.P. (1996). Stimulation of root hair elongation in Arabidopsis thaliana by low phosphorus availability. Plant Cell Environ.

[31] Brown L.K., George T.S., Dupuy L.X., White P.J. (2012). A conceptual model of root hair ideotypes for future agricultural environments: What combination of traits should be targeted to cope with limited P availability?. Ann. Bot..

[32] Ma Z., Bielenberg D.G., Brown K.M., Lynch J.P. (2001). Regulation of root hair density by phosphorus availability in Arabidopsis thaliana. Plant Cell Environ.

[33] Lopez-Bucio J., Cruz-Ramirez A., Herrera-Estrella L. (2003). The role of nutrient availability in regulating root architecture. Curr. Opin. Plant Biol.

[34] Peret B., Clement M., Nussaume L., Desnos T. (2011). Root developmental adaptation to phosphate starvation: Better safe than sorry. Trends Plant Sci.

[35] Drew M.C., Saker L.R. (1978). Nutrient supply and growth of seminal root-system in barley. III. Compensatory increases in growth of lateralroots, and in rates of phosphate uptake, in response to a localized supply of phosphate. J. Exp. Bot.

[36] Jackson R.B., Manwaring J.H., Caldwell M.M. (1990). Rapid physiological adjustment of roots to localized soil enrichment. Nature.

[37] Smith S.E., Smith F.A., Jakobsen I. (2003). Mycorrhizal fungi can dominate phosphate supply to plants irrespective of growth responses. Plant Physiol.

[38] Bonfante P., Anca I.A. (2009). Plants, mycorrhizal fungi, and bacteria: A network of interactions. Annu. Rev. Microbiol.

[39] Purnell H. (1960). Studies of the family proteaceae. I. Anatomy and morphology of the roots of some victorian species. Aust. J. Bot.

[40] Gardner W.K., Barber D.A., Parbery D.G. (1982). Effect of microorganisms on the formation and activity of proteoid roots of *Lupinus albus* L. Aust. J. Bot.

[41] Johnson J.F., Allan D.L., Vance C.P. (1994). Phosphorus stress-induced proteoid roots show altered metabolism in *Lupinus albus*. Plant Physiol.

[42] Malajczu N., Bowen G.D. (1974). Proteoid roots are microbially induced. Nature.

[43] Watt M., Evans J.R. (1999). Proteoid roots. Physiology and development. Plant Physiol.

[44] Jones D.L. (1998). Organic acids in the rhizosphere—A critical review. Plant Soil.

[45] Li M.G., Osaki M., Rao I.M., Tadano T. (1997). Secretion of phytase from the roots of several plant species under phosphorus-deficient conditions. Plant Soil.

[46] Wang X.R., Wang Y.X., Tian J., Lim B.L., Yan X.L., Liao H. (2009). Overexpressing AtPAP15 enhances phosphorus efficiency in soybean. Plant Physiol.

[47] Neumann G., George T.S., Plassard C. (2009). Strategies and methods for studying the rhizosphere-the plant science toolbox. Plant Soil.

[48] Duff S.M.G., Sarath G., Plaxton W.C. (1994). The role of acid-phosphatases in plant phosphorus-metabolism. Physiol. Plant.

[49] Tadano T., Sakai H. (1991). Secretion of acid-phosphatase by the roots of several crop species under phosphorus-deficient conditions. Soil Sci. Plant Nutr.

[50] Starnes D.L., Padmanabhan P., Sahi S.V. (2008). Effect of P sources on growth, P accumulation and activities of phytase and acid phosphatases in two cultivars of annual ryegrass (*Lolium multiflorum* L.). Plant Physiol. Biochem.

[51] Plaxton W.C., Tran H.T. (2011). Metabolic adaptations of phosphate-starved plants. Plant Physiol.

[52] Ai P.H., Sun S.B., Zhao J.N., Fan X.R., Xin W.J., Guo Q., Yu L., Shen Q.R., Wu P., Miller A.J. (2009). Two rice phosphate transporters, OsPht1;2 and OsPht1;6, have different functions and kinetic properties in uptake and translocation. Plant J.

[53] Shin H., Shin H.S., Dewbre G.R., Harrison M.J. (2004). Phosphate transport in Arabidopsis: Pht1;1 and Pht1; 4 play a major role in phosphate acquisition from both low- and high-phosphate environments. Plant J.

[54] Daram P., Brunner S., Rausch C., Steiner C., Amrhein N., Bucher M. (1999). Pht2;1 encodes a low-affinity phosphate transporter from Arabidopsis. Plant Cell.

[55] Poirier Y., Bucher M. (2002). Phosphate transport and homeostasis in Arabidopsis. The Arabidopsis B.

[56] Tu S.I., Cavanaugh J.R., Boswell R.T. (1990). Phosphate-uptake by excised maize root-tips studied by *in vivo* P-31 nuclear-magnetic-resonance spectroscopy. Plant Physiol.

[57] Pratt J., Boisson A.M., Gout E., Bligny R., Douce R., Aubert S. (2009). Phosphate (Pi) starvation effect on the cytosolic Pi concentration and Pi exchanges across the tonoplast in plant cells: An *in vivo* P-31-nuclear magnetic resonance study using methylphosphonate as a Pi analog. Plant Physiol.

[58] Duff S.M.G., Moorhead G.B.G., Lefebvre D.D., Plaxton W.C. (1989). Phosphate starvation inducible bypasses of adenylate and phosphate dependent glycolytic-enzymes in Brassica nigra suspension cells. Plant Physiol.

[59] Rychter A.M., Mikulska M. (1990). The relationship between phosphate status and cyanide-resistant respiration in bean roots. Physiol. Plant.

[60] Hartel H., Dormann P., Benning C. (2000). DGD1-independent biosynthesis of extraplastidic galactolipids after phosphate deprivation in Arabidopsis. Proc. Natl. Acad. Sci. USA.

[61] Stitt M. (1998). Pyrophosphate as an energy donor in the cytosol of plant cells: An enigmatic alternative to ATP. Bot. Acta.

[62] Weiner H., Stitt M., Heldt H.W. (1987). Subcellular compartmentation of pyrophosphate and alkaline pyrophosphatase in leaves. Biochim. Biophys. Acta.

[63] Chiou T.J., Lin S.I. (2011). Signaling network in sensing phosphate availability in plants. Annu. Rev. Plant Biol.

[64] Hu B., Chu C. (2011). Phosphate starvation signaling in rice. Plant Signal. Behav.

[65] Yang X.J., Finnegan P.M. (2010). Regulation of phosphate starvation responses in higher plants. Ann. Bot.

[66] Rouached H., Arpat A.B., Poirier Y. (2010). Regulation of phosphate starvation responses in plants: Signaling players and cross-talks. Mol. Plant.

[67] Yuan H., Liu D. (2008). Signaling components involved in plant responses to phosphate starvation. J. Integr. Plant Biol.

[68] Ticconi C.A., Delatorre C.A., Abel S. (2001). Attenuation of phosphate starvation responses by phosphite in arabidopsis. Plant Physiol.

[69] Varadarajan D.K., Karthikeyan A.S., Matilda P.D., Raghothama K.G. (2002). Phosphite, an analog of phosphate, suppresses the coordinated expression of genes under phosphate starvation. Plant Physiol.

[70] Linkohr B.I., Williamson L.C., Fitter A.H., Leyser H.M.O. (2002). Nitrate and phosphate availability and distribution have different effects on root system architecture of Arabidopsis. Plant J.

[71] Svistoonoff S., Creff A., Reymond M., Sigoillot-Claude C., Ricaud L., Blanchet A., Nussaume L., Desnos T. (2007). Root tip contact with low-phosphate media reprograms plant root architecture. Nat. Genet.

[72] Ticconi C.A., Delatorre C.A., Lahner B., Salt D.E., Abel S. (2004). Arabidopsis pdr2 reveals a phosphate-sensitive checkpoint in root development. Plant J.

[73] Ticconi C.A., Lucero R.D., Sakhonwasee S., Adamson A.W., Creff A., Nussaume L., Desnos T., Abel S. (2009). ER-resident proteins PDR2 and LPR1 mediate the developmental response of root meristems to phosphate availability. Proc. Natl. Acad. Sci. USA.

[74] Sanchez-Calderon L., Lopez-Bucio J., Chacon-Lopez A., Gutierrez-Ortega A., Hernandez-Abreu E., Herrera-Estrella L. (2006). Characterization of low phosphorus insensitive mutants reveals a crosstalk between low phosphorus-induced determinate root development and the activation of genes involved in the adaptation of Arabidopsis to phosphorus deficiency. Plant Physiol.

[75] Mayzlish-Gati E., De-Cuyper C., Goormachtig S., Beeckman T., Vuylsteke M., Brewer P.B., Beveridge C.A., Yermiyahu U., Kaplan Y., Enzer Y. (2012). Strigolactones are involved in root response to low phosphate conditions in Arabidopsis. Plant Physiol.

[76] Perez-Torres C.A., Lopez-Bucio J., Cruz-Ramirez A., Ibarra-Laclette E., Dharmasiri S., Estelle M., Herrera-Estrella L. (2008). Phosphate availability alters lateral root development in Arabidopsis by modulating auxin sensitivity via a mechanism involving the TIR1 auxin receptor. Plant Cell.

[77] Gilbert G.A., Knight J.D., Vance C.P., Allan D.L. (2000). Proteoid root development of phosphorus deficient lupin is mimicked by auxin and phosphonate. Ann. Bot.

[78] Franco-Zorrilla J.M., Martin A.C., Leyva A., Par-Ares J.P. (2005). Interaction between phosphate-starvation, sugar, and cytokinin signaling in Arabidopsis and the roles of cytokinin receptors CRE1/AHK4 and AHK3. Plant Physiol.

[79] Jiang C.F., Gao X.H., Liao L., Harberd N.P., Fu X.D. (2007). Phosphate starvation root architecture and anthocyanin accumulation responses are modulated by the gibberellin-DELLA signaling pathway in Arabidopsis. Plant Physiol.

[80] Gibson S.I. (2004). Sugar and phytohormone response pathways: navigating a signalling network. J. Exp. Bot.

[81] Kircher S., Schopfer P. (2012). Photosynthetic sucrose acts as cotyledon-derived long-distance signal to control root growth during early seedling development in Arabidopsis. Proc. Natl. Acad. Sci. USA.

[82] Aung K., Lin S.I., Wu C.C., Huang Y.T., Su C.L., Chiou T.J. (2006). pho2, a phosphate overaccumulator, is caused by a nonsense mutation in a microRNA399 target gene. Plant Physiol.

[83] Chiou T.J., Aung K., Lin S.I., Wu C.C., Chiang S.F., Su C.L. (2006). Regulation of phosphate homeostasis by microRNA in Arabidopsis. Plant Cell.

[84] Bari R., Datt Pant B., Stitt M., Scheible W.R. (2006). PHO2, microRNA399, and PHR1 define a phosphate-signaling pathway in plants. Plant Physiol.

[85] Pant B.D., Buhtz A., Kehr J., Scheible W.R. (2008). MicroRNA399 is a long-distance signal for the regulation of plant phosphate homeostasis. Plant J.

[86] Lin S.I., Chiang S.F., Lin W.Y., Chen J.W., Tseng C.Y., Wu P.C., Chiou T.J. (2008). Regulatory network of microRNA399 and PHO2 by systemic signaling. Plant Physiol.

[87] Matusova R., Rani K., Verstappen F.W.A., Franssen M.C.R., Beale M.H., Bouwmeester H.J. (2005). The strigolactone germination stimulants of the plant-parasitic Striga and Orobanche spp. are derived from the carotenoid pathway. Plant Physiol.

[88] Alder A., Jamil M., Marzorati M., Bruno M., Vermathen M., Bigler P., Ghisla S., Bouwmeester H., Beyer P., Al-Babili S. (2012). The path from beta-carotene to carlactone, a strigolactone-like plant hormone. Science.

[89] Cook C.E., Whichard L.P., Turner B., Wall M.E. (1966). Germination of witchweed (Striga lutea Lour.)—Isolation and properties of a potent stimulant. Science.

[90] Besserer A., Puech-Pages V., Kiefer P., Gomez-Roldan V., Jauneau A., Roy S., Portais J.C., Roux C., Becard G., Sejalon-Delmas N. (2006). Strigolactones stimulate arbuscular mycorrhizal fungi by activating mitochondria. PLoS Biol.

[91] Kohlen W., Charnikhova T., Liu Q., Bours R., Domagalska M.A., Beguerie S., Verstappen F., Leyser O., Bouwmeester H., Ruyter-Spira C. (2011). Strigolactones are transported through the xylem and play a key role in shoot architectural response to phosphate deficiency in nonarbuscular mycorrhizal host Arabidopsis. Plant Physiol.

[92] Kretzschmar T., Kohlen W., Sasse J., Borghi L., Schlegel M., Bachelier J.B., Reinhardt D., Bours R., Bouwmeester H.J., Martinoia E. (2012). A petunia ABC protein controls strigolactone-dependent symbiotic signalling and branching. Nature.

[93] Beveridge C.A., Kyozuka J. (2010). New genes in the strigolactone-related shoot branching pathway. Curr. Opin. Plant Biol.

[94] Domagalska M.A., Leyser O. (2011). Signal integration in the control of shoot branching. Nat. Rev. Mol. Cell Biol.

[95] Tsuchiya Y., McCourt P. (2012). Strigolactones as small molecule communicators. Mol. Biosystems.

[96] Xie X.N., Yoneyama K., Yoneyama K. (2010). The strigolactone story. Annu. Rev. Phytopathol.

[97] Koltai H. (2011). Strigolactones are regulators of root development. New Phytol.

[98] Wang Y.H., Li J.Y. (2011). Branching in rice. Curr. Opin. Plant Biol.

[99] Seto Y., Kameoka H., Yamaguchi S., Kyozuka J. (2012). Recent advances in strigolactone research: Chemical and biological aspects. Plant Cell Physiol.

[100] Ruyter-Spira C., Al-Babili S., van der Krol S., Bouwmeester H. (2012). The biology of strigolactones. Trends Plant Sci.

[101] Brewer P.B., Koltai H., Beveridge C.A. (2013). Diverse roles of strigolactones in plant development. Mol. Plant.

[102] Booker J., Auldridge M., Wills S., McCarty D., Klee H., Leyser O. (2004). MAX3/CCD7 is a carotenoid cleavage dioxygenase required for the synthesis of a novel plant signaling molecule. Curr. Biol.

[103] Sorefan K., Booker J., Haurogne K., Goussot M., Bainbridge K., Foo E., Chatfield S., Ward S., Beveridge C., Rameau C. (2003). MAX4 and RMS1 are orthologous dioxygenase-like genes that regulate shoot branching in Arabidopsis and pea. Genes Dev.

[104] Booker J., Sieberer T., Wright W., Williamson L., Willett B., Stirnberg P., Turnbull C., Srinivasan M., Goddard P., Leyser O. (2005). MAX1 encodes a cytochrome P450 family member that acts downstream of MAX3/4 to produce a carotenoid-derived branch-inhibiting hormone. Dev. Cell.

[105] Snowden K.C., Simkin A.J., Janssen B.J., Templeton K.R., Loucas H.M., Simons J.L., Karunairetnam S., Gleave A.P., Clark D.G., Klee H.J. (2005). The Decreased apical dominance 1/petunia hybrida carotenoid cleavage dioxygenase 8 gene affects branch production and plays a role in leaf senescence, root growth, and flower development. Plant Cell.

[106] Lin H., Wang R.X., Qian Q., Yan M.X., Meng X.B., Fu Z.M., Yan C.Y., Jiang B., Su Z., Li J.Y. (2009). DWARF27, an iron-containing protein required for the biosynthesis of strigolactones, regulates rice tiller bud outgrowth. Plant Cell.

[107] Waters M.T., Brewer P.B., Bussell J.D., Smith S.M., Beveridge C.A. (2012). The Arabidopsis ortholog of rice DWARF27 acts upstream of MAX1 in the control of plant development by strigolactones. Plant Physiol.

[108] Liu W., Kohlen W., Lillo A., Op den Camp R., Ivanov S., Hartog M., Limpens E., Jamil M., Smaczniak C., Kaufmann K. (2011). Strigolactone biosynthesis in Medicago truncatula and rice requires the symbiotic GRAS-type transcription factors NSP1 and NSP2. Plant Cell.

[109] Stirnberg P., van de Sande K., Leyser H.M.O. (2002). MAX1 and MAX2 control shoot lateral branching in Arabidopsis. Development.

[110] Arite T., Umehara M., Ishikawa S., Hanada A., Maekawa M., Yamaguchi S., Kyozuka J. (2009). d14, a strigolactone-insensitive mutant of rice, shows an accelerated outgrowth of tillers. Plant Cell Physiol.

[111] Waters M.T., Nelson D.C., Scaffidi A., Flematti G.R., Sun Y.K.M., Dixon K.W., Smith S.M. (2012). Specialisation within the DWARF14 protein family confers distinct responses to karrikins and strigolactones in Arabidopsis. Development.

[112] Hamiaux C., Drummond R.S.M., Janssen B.J., Ledger S.E., Cooney J.M., Newcomb R.D., Snowden K.C. (2012). DAD2 is an alpha/beta hydrolase likely to be involved in the perception of the plant branching hormone, strigolactone. Curr. Biol.

[113] Santner A., Estelle M. (2009). Recent advances and emerging trends in plant hormone signalling. Nature.

[114] Ueguchi-Tanaka M., Ashikari M., Nakajima M., Itoh H., Katoh E., Kobayashi M., Chow T.Y., Hsing Y.I.C., Kitano H., Yamaguchi I. (2005). GIBBERELLIN INSENSITIVE DWARF1 encodes a soluble receptor for gibberellin. Nature.

[115] Gaiji N., Cardinale F., Prandi C., Bonfante P., Ranghino G. (2012). The computational-based structure of Dwarf14 provides evidence for its role as potential strigolactone receptor in plants. BMC Res. Notes.

[116] Zhao L.H., Zhou X.E., Wu Z.S., Yi W., Xu Y., Li S., Xu T.H., Liu Y., Chen R.Z., Kovach A. (2013). Crystal structures of two phytohormone signal-transducing alpha/beta hydrolases: Karrikin-signaling KAI2 and strigolactone-signaling DWARF14. Cell Res.

[117] Kagiyama M., Hirano Y., Mori T., Kim S.Y., Kyozuka J., Seto Y., Yamaguchi S., Hakoshima T. (2013). Structures of D14 and D14L in the strigolactone and karrikin signaling pathways. Genes Cells.

[118] Minakuchi K., Kameoka H., Yasuno N., Umehara M., Luo L., Kobayashi K., Hanada A., Ueno K., Asami T., Yamaguchi S. (2010). FINE CULM1 (FC1) works downstream of strigolactones to inhibit the outgrowth of axillary buds in rice. Plant Cell Physiol.

[119] Takeda T., Suwa Y., Suzuki M., Kitano H., Ueguchi-Tanaka M., Ashikari M., Matsuoka M., Ueguchi C. (2003). The OsTB1 gene negatively regulates lateral branching in rice. Plant J.

[120] Doebley J., Stec A., Hubbard L. (1997). The evolution of apical dominance in maize. Nature.

[121] Aguilar-Martinez J.A., Poza-Carrion C., Cubas P. (2007). Arabidopsis BRANCHED1 acts as an integrator of branching signals within axillary buds. Plant Cell.

[122] Braun N., de Saint Germain A., Pillot J.P., Boutet-Mercey S., Dalmais M., Antoniadi I., Li X., Maia-Grondard A., Le Signor C., Bouteiller N. (2012). The pea TCP transcription factor PsBRC1 acts downstream of strigolactones to control shoot branching. Plant Physiol.

[123] Breuillin F., Schramm J., Hajirezaei M., Ahkami A., Favre P., Druege U., Hause B., Bucher M., Kretzschmar T., Bossolini E. (2010). Phosphate systemically inhibits development of arbuscular mycorrhiza in Petunia hybrida and represses genes involved in mycorrhizal functioning. Plant J.

[124] Balzergue C., Puech-Pages V., Becard G., Rochange S.F. (2011). The regulation of arbuscular mycorrhizal symbiosis by phosphate in pea involves early and systemic signalling events. J. Exp. Bot.

[125] Foo E., Yoneyama K., Hugill C.J., Quittenden L.J., Reid J.B. (2013). Strigolactones and the regulation of pea symbioses in response to nitrate and phosphate deficiency. Mol. Plant.

[126] Ruyter-Spira C., Kohlen W., Charnikhova T., van Zeijl A., van Bezouwen L., de Ruijter N., Cardoso C., Lopez-Raez J.A., Matusova R., Bours R. (2011). Physiological effects of the synthetic strigolactone analog GR24 on root system architecture in Arabidopsis: another belowground role for strigolactones?. Plant Physiol.

[127] Kapulnik Y., Delaux P.M., Resnick N., Mayzlish-Gati E., Wininger S., Bhattacharya C., Sejalon-Delmas N., Combier J.P., Becard G., Belausov E. (2011). Strigolactones affect lateral root formation and root-hair elongation in Arabidopsis. Planta.

[128] Rasmussen A., Mason M.G., De Cuyper C., Brewer P.B., Herold S., Agusti J., Geelen D., Greb T., Goormachtig S., Beeckman T. (2012). Strigolactones suppress adventitious rooting in Arabidopsis and Pea. Plant Physiol.

[129] Lopez-Raez J.A., Bouwmeester H. (2008). Fine-tuning regulation of strigolactone biosynthesis under phosphate starvation. Plant Signal. Behav.

[130] Yoneyama K., Xie X.N., Sekimoto H., Takeuchi Y., Ogasawara S., Akiyama K., Hayashi H., Yoneyama K. (2008). Strigolactones, host recognition signals for root parasitic plants and arbuscular mycorrhizal fungi, from Fabaceae plants. New Phytol.

[131] Lopez-Raez J.A., Charnikhova T., Gomez-Roldan V., Matusova R., Kohlen W., De Vos R., Verstappen F., Puech-Pages V., Becard G., Mulder P. (2008). Tomato strigolactones are derived from carotenoids and their biosynthesis is promoted by phosphate starvation. New Phytol.

[132] Jamil M., Charnikhova T., Cardoso C., Jamil T., Ueno K., Verstappen F., Asami T., Bouwmeester H.J. (2011). Quantification of the relationship between strigolactones and Striga hermonthica infection in rice under varying levels of nitrogen and phosphorus. Weed Res.

[133] Yoneyama K., Xie X.N., Kim H.I., Kisugi T., Nomura T., Sekimoto H., Yokota T., Yoneyama K. (2012). How do nitrogen and phosphorus deficiencies affect strigolactone production and exudation?. Planta.

[134] Yoneyama K., Xie X.N., Kisugi T., Nomura T., Sekimoto H., Yokota T., Yoneyama K. (2011). Characterization of strigolactones exuded by Asteraceae plantss. Plant Growth Regul.

[135] Smith S.E., Read D.J. (2008). Mycorrhizal Symbiosis.

[136] Harrison M.J. (1999). Molecular and cellular aspects of the arbuscular mycorrhizal symbiosis. Annu. Rev. Plant Physiol.

[137] Nagahashi G., Douds D.D. (2000). Partial separation of root exudate components and their effects upon the growth of germinated spores of AM fungi. Mycol. Res.

[138] Besserer A., Becard G., Jauneau A., Roux C., Sejalon-Delmas N. (2008). GR24, a synthetic analog of strigolactones, stimulates the mitosis and growth of the arbuscular mycorrhizal fungus Gigaspora rosea by boosting its energy metabolism. Plant Physiol.

[139] Yoshida S., Kameoka H., Tempo M., Akiyama K., Umehara M., Yamaguchi S., Hayashi H., Kyozuka J., Shirasu K. (2012). The D3 F-box protein is a key component in host strigolactone responses essential for arbuscular mycorrhizal symbiosis. New Phytol.

[140] Kang J., Hwang J.U., Lee M., Kim Y.Y., Assmann S.M., Martinoia E., Lee Y. (2010). PDR-type ABC transporter mediates cellular uptake of the phytohormone abscisic acid. Proc. Natl. Acad. Sci. USA.

[141] Woo H.R., Chung K.M., Park J.H., Oh S.A., Ahn T., Hong S.H., Jang S.K., Nam H.G. (2001). ORE9, an F-box protein that regulates leaf senescence in Arabidopsis. Plant Cell.

[142] Yan H., Saika H., Maekawa M., Takamure I., Tsutsumi N., Kyozuka J., Nakazono M. (2007). Rice tillering dwarf mutant dwarf3 has increased leaf longevity during darkness-induced senescence or hydrogen peroxide-induced cell death. Genes Genet. Syst.

[143] Crafts-Brandner S.J. (1992). Phosphorus nutrition influence on leaf senescence in soybean. Plant Physiol.

